# Non-Coding RNAs as Biomarkers and Therapeutic Targets for Diabetic Kidney Disease

**DOI:** 10.3389/fphar.2020.583528

**Published:** 2021-01-26

**Authors:** Yue-Yu Gu, Fu-Hua Lu, Xiao-Ru Huang, Lei Zhang, Wei Mao, Xue-Qing Yu, Xu-Sheng Liu, Hui-Yao Lan

**Affiliations:** ^1^ Department of Nephrology and State Key Laboratory of Dampness Syndrome of Chinese Medicine, Guangdong Provincial Hospital of Chinese Medicine, The Second Affiliated Hospital, Guangzhou University of Chinese Medicine, Guangzhou, China; ^2^ Department of Medicine and Therapeutics, Li Ka Shing Institute of Health Sciences, The Chinese University of Hong Kong, Hong Kong, China; ^3^ Guangdong-Hong Kong Joint Laboratory for Immunological and Genetic Kidney Diseases, Guangdong Academy of Medical Sciences, Guangdong Provincial People’s Hospital, Guangzhou, China; ^4^ Guangdong-Hong Kong Joint Laboratory for Immunological and Genetic Kidney Diseases, The Chinese University of Hong Kong, Hong Kong, China

**Keywords:** diabetic kidney disease, micro RNAs, long non-coding RNAs, TGF-β, fibrosis, inflammation, biomarker, therapeutic target

## Abstract

Diabetic kidney disease (DKD) is the most common diabetic complication and is a leading cause of end-stage kidney disease. Increasing evidence shows that DKD is regulated not only by many classical signaling pathways but also by epigenetic mechanisms involving chromatin histone modifications, DNA methylation, and non-coding RNA (ncRNAs). In this review, we focus on our current understanding of the role and mechanisms of ncRNAs, including microRNAs (miRNAs) and long non-coding RNAs (lncRNAs) in the pathogenesis of DKD. Of them, the regulatory role of TGF-β/Smad3-dependent miRNAs and lncRNAs in DKD is highlighted. Importantly, miRNAs and lncRNAs as biomarkers and therapeutic targets for DKD are also described, and the perspective of ncRNAs as a novel therapeutic approach for combating diabetic nephropathy is also discussed.

## Introduction

Diabetic kidney disease (DKD) is one of the most predominant diabetic complications and is a leading cause of chronic kidney disease (CKD). It is reported that up to 20–50% of living diabetes, including type 1 (T1DM) and type 2 (T2DM) diabetes, would eventually develop into DKD (Selby and Taal, 2020), which contributes to the high mortality of patients with DKD (Braunwald, 2019). The established DKD is characterized by the onset of persistent albuminuria and progressive decline of estimated glomerular filtration rate (eGFR) (Magee et al., 2017). Pathologically, the histological features of DKD include the thickening of the glomerular basement membrane (GBM), glomerular capillary hypertension, mesangial expansion, nodular sclerosis, glomerulosclerosis, interstitial fibrosis, inflammation, and tubular atrophy (Raval et al., 2020).

**FIGURE 1:**
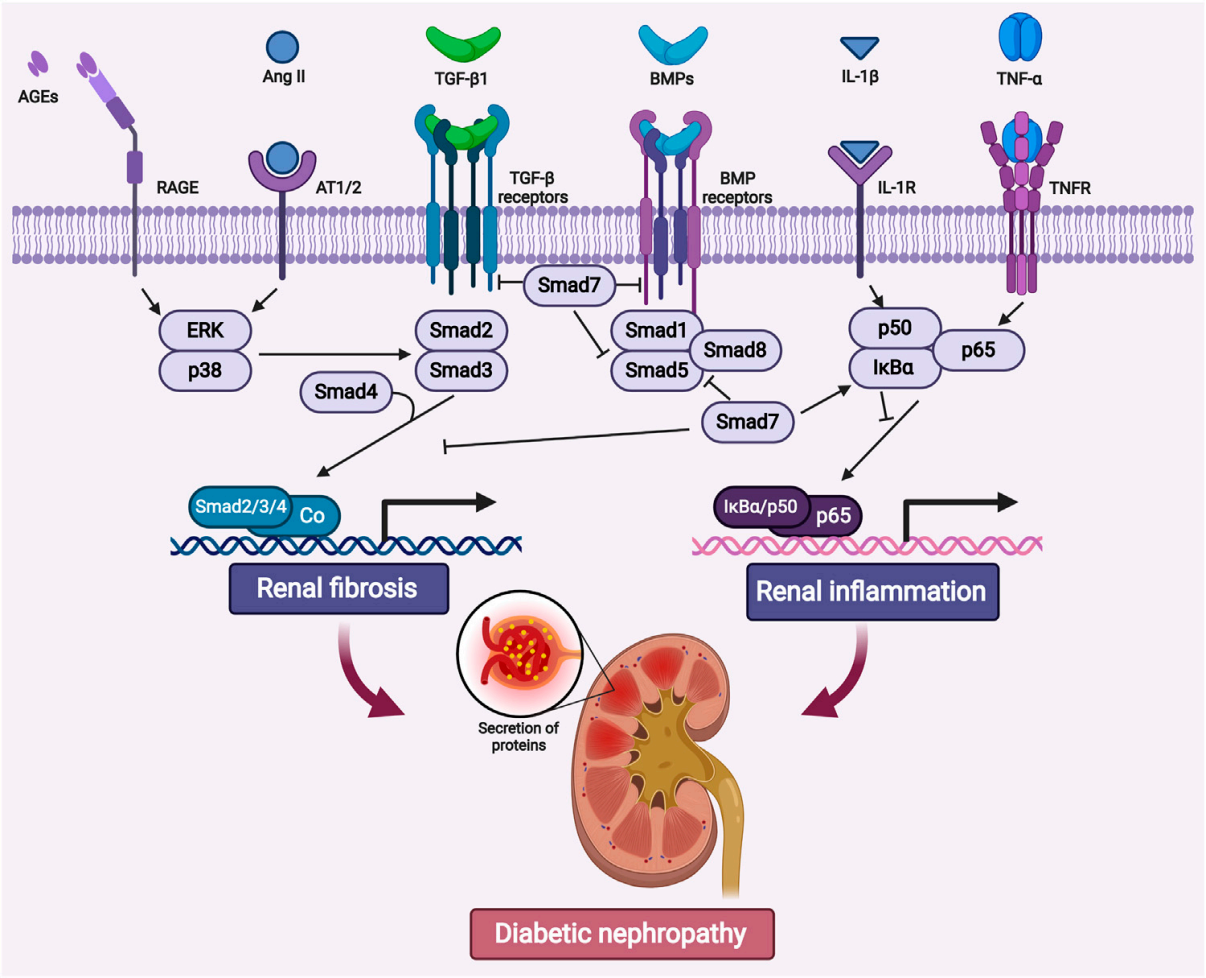
The crosstalk of canonical and noncanonical TGF-β signaling pathways associated with renal fibrosis and inflammation in diabetic nephropathy. TGF-β/Smad and NF-κB signaling pathway are highly activated under hyperglycemic conditions. AGEs, Ang II, IL-1β and TNF-α etc, may trigger these two pathways to promote fibrosis and inflammation in diabetic kidneys. Abbreviations: AGEs, advanced glycation end products; RAGE, receptor for AGE; Ang II, angiotensin II; AT1/2, Ang II receptor 1 and 2; BMP, bone morphogenic protein; TNF-α, tumor necrosis factor α; TNFR, TNF receptor; IL-1β, interleukin 1β; IL-1R, IL-1 receptor; ERK, extracellular-signal regulated kinase; IκBα, nuclear factor of kappa light polypeptide gene enhancer in B-cells inhibitor α. (Figure created with BioRender.com).

In patients with diabetes, hyperglycemia may trigger oxidative stress, renal inflammation, and fibrosis in kidneys (Matoba et al., 2019; Patel et al., 2020). Among those pathogenic factors, renal fibrogenesis is the major driving force in the development of DKD (Hills and Squires, 2011; Lan, 2012a). It is well-established that transforming growth factor β (TGF-β) as the master regulator for the fibrotic and inflammatory process in CKD (Meng et al., 2016). Hyperglycemic factors such as advanced glycation end products (AGEs) and angiotensin II (AngII) may trigger the activation of TGF-β signaling *via* Smad dependent or independent pathway, therefore promoting fibrosis in kidneys (Lan, 2011; Meng et al., 2016; Gu et al., 2020) (Figure 1).

The emerging field of epigenetic regulation by ncRNAs has focused on the pathogenic pathways to halt the progression of DKD. With no function in protein-coding, ncRNAs were implicated as therapeutic targets or biomarkers for DKD (Loganathan et al., 2020). Interestingly, these ncRNAs could also be regulated by TGF-β (Meng et al., 2015). In this review, we will focus on the regulatory role of miRNAs and lncRNAs in the progression of DKD, and their potentials as therapeutic targets and biomarkers for DKD are highlighted. Moreover, the mechanisms of ncRNAs on renal fibrosis and inflammation in DKD based on the TGF-β/Smad-mediated signaling pathway will also be discussed.

## The Emerging Role of Non-coding RNAs in DKD

**FIGURE 2:**
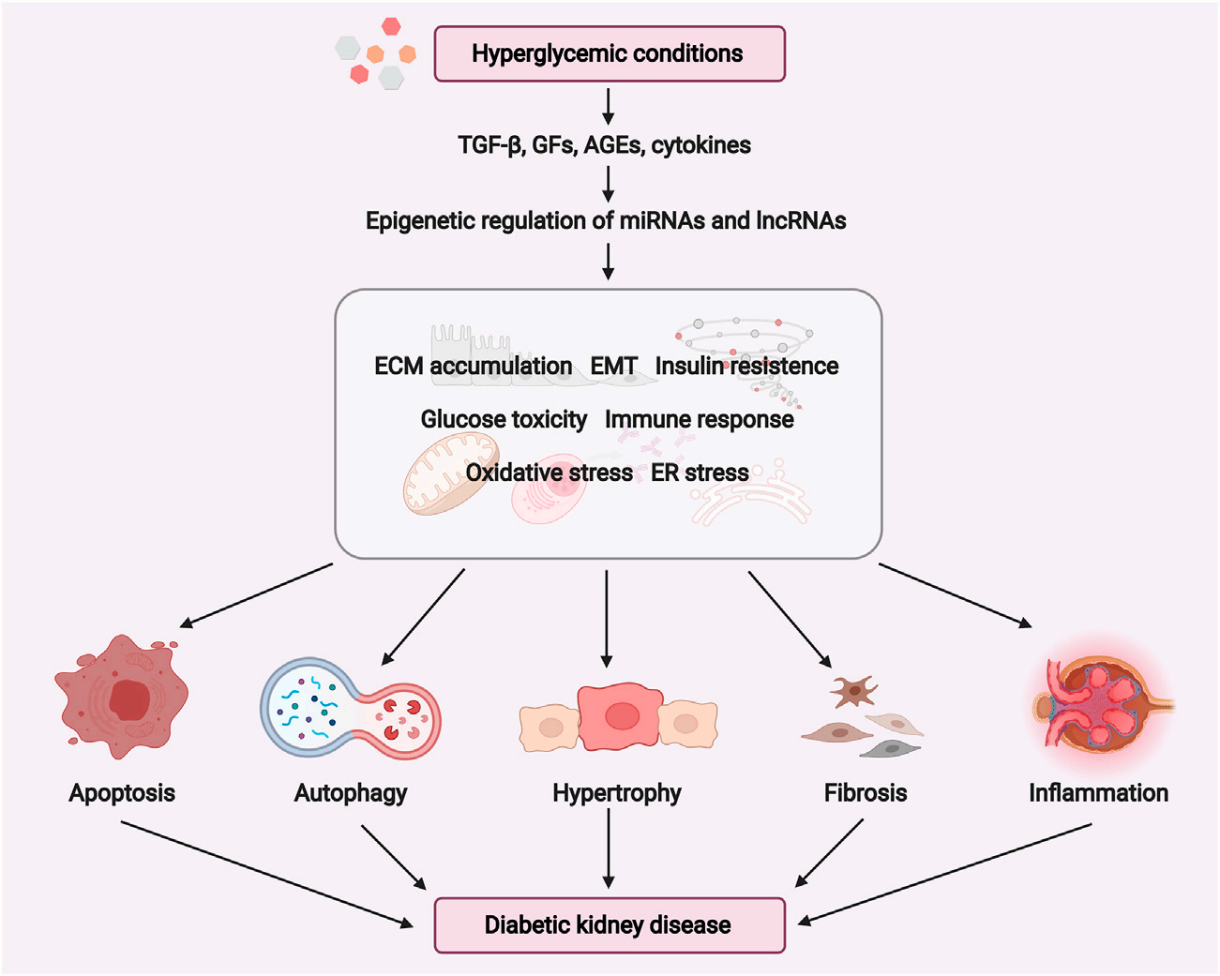
Potential role of miRNAs and lncRNAs in the pathogenesis of diabetic kidney disease. Under hyperglycemic conditions, the expression of TGF-β, growth factors such as CTGF, FGF, and cytokines may induce ECM accumulation, EMT, ER stress, oxidative stress, insulin resistance, glucose toxicity, fibrosis, and inflammatory response. These pathogenic processes are positively or negatively regulated by ncRNAs (miRNAs and lncRNAs) to promote cell apoptosis, autophagy, hypertrophy, fibrosis, inflammation in the diabetic kidney. Abbreviations: GFs, growth factors; ECM, extracellular matrix; EMT, epithelial-mesenchymal transition; ER, endoplasmic reticulum. (Figure created with BioRender.com).

miRNAs are single-stranded endogenous RNAs (20–22 nucleotides in length) that regulate gene expression on the post-transcriptional or transcriptional level (Wahid et al., 2010). LncRNAs are RNA transcripts over 200 nucleotides in length, which are able to modulate gene expression by binding to either DNAs, RNAs, or proteins (Yao et al., 2019). The roles of miRNAs and lncRNAs in kidney development and disease have been reviewed (Kaucsár et al., 2010; Moghaddas Sani et al., 2018; Zhou et al., 2019). Thus, we mainly focus on the roles and underlying mechanisms of miRNAs and lncRNAs relevant to DKD pathogenesis (as shown in Figure 2).

### Non-Smad-dependent miRNAs in DKD

The functional relevance of miRNA in renal diseases has caught our attention since the rapid development of RNA sequencing strategy. In most cases, miRNAs hybridize to the 3’UTRs (untranslated regions) of the target mRNAs and hence silencing the expression of target genes. Up to date, the function and underlying mechanisms of many miRNAs in renal diseases have been well-demonstrated and reviewed (Hou and Zhao, 2013). These miRNAs are of great importance to the epigenetic regulation on DKD.

Renal tubulointerstitial fibrosis (TIF) is one of the predominant features of DKD. A group of miRNAs have been shown to be profibrotic in DKD **(** Table 1). The expression of miR-22 was increased in streptozotocin (STZ)-induced DKD model and in high glucose (HG)-treated tubular epithelial cells (TECs). miR-22 targets phosphatase and tensin homolog (PTEN), therefore suppressing autophagy and inducing the expression of collagen IV and α-smooth muscle actin (α-SMA) (Zhang et al., 2018). A high level of miR-23a was also observed in diabetic patients and HG-cultured TECs. It directly targets the nuclear transcription co-repressor Ski-related novel protein N (SnoN) (Tan et al., 2006), a crucial negative regulator to TGF-β/Smad3-mediated signaling pathway, to induce fibrosis in DKD (Xu et al., 2018a). Sirtuin 1 (SIRT1) expression in the nucleus and the cytoplasm has also been shown as a renoprotective regulator by inhibiting TGF-β/Smad-induced fibrosis and downstream hypoxia-inducible factor-1α (HIF-1α). miR-34a-5p, miR-217, miR-133b, and miR-199b may dcirectly or indirectly target and suppress the expression of SIRT1 under hyperglycemic conditions (Shao et al., 2016; Sun et al., 2018b; Xue et al., 2018). The transient receptor potential cation channel subfamily C member 1 (TRPC1) is downregulated in diabetic patients and animal models, which may contribute to the development of DKD (Zhang et al., 2009a). miR-135a targets TRPC1 to promote the fibrotic process in diabetic renal injury (He et al., 2014). Interestingly, diabetic-induced albumin triggers the expression of miR-184 in the tubular cells to promote TIF, which is associated with decreased expression of lipid phosphate phosphatase 3 (LPP3) (Zanchi et al., 2017). The canopy 1 (CNPY1) is a target of miR-370 to modulate fibroblast growth element signaling (Matsui et al., 2011). Overexpression of miR-370 significantly increases the accumulation of extracellular matrix (ECM) and promotes the proliferation of mesangial cells (MCs) (Yu et al., 2019). On the other hand, the anti-fibrotic miR-342 binds to the 3’UTR of SRY-box 6 (SOX6), therefore inhibiting SOX6 expression and the level of fibrotic biomarkers (Jiang et al., 2020b). miR-379 is also involved in the pathogenesis of DKD. It is reported that miR-379 triggers miR-let-7, which prevents ECM accumulation and proliferation of MCs (Li et al., 2019b). Nevertheless, some miRNAs exert protective effects by inhibiting the epithelial-to-mesenchymal transition (EMT). Notably, miR-30c, miR-98-5p and miR-302a-3p target the fibrosis-related JAK1, Snail1, HMGA2, and ZEB1, respectively, thus blocking the fibrotic process in DKD by inhibiting EMT (Zhao et al., 2017; Tang et al., 2018b; Zhu et al., 2019c; Gao et al., 2020). Furthermore, miR-455-3p also inhibits renal fibrosis by targeting ROCK2, together with the reduction of anti-inflammatory cytokines such as tumor necrosis factor-α (TNF-α) and monocyte chemotactic protein 1 (MCP-1) (Wu et al., 2018a). Interestingly, miR-455-3p also serves as a sponge for pathogenic lncRNA Hottip. Hottip is upregulated under HG conditions, while miR-455-3p may reverse Hottip-mediated fibrosis and inflammation (Zhu et al., 2019b). Fatty acid accumulation (FAC) was also induced by DKD, fatty acid synthase (FASN) is not only the vital lipogenic enzyme to FAC, but also an upregulated molecule that contributes to glomerulosclerosis and renal inflammation. miR-544 binds to the 3’UTR of FASN thus attenuating the infiltration of inflammatory cells, the activation of NF-κB signaling and renal fibrosis (Sun et al., 2020). All these findings have suggested a crucial role of miRNAs in DKD-induced renal fibrosis based on the epigenetic regulation level.

**TABLE 1:** Non-Smad-dependent miRNAs in DKD.

miRNA	Target	Pathological output(s)	References
miR-22	PTEN	Pro-fibrosis	(Zhang et al., 2018)
miR-23a	SnoN		(Xu et al., 2018a)
miR-34a-5p	SIRT1		(Xue et al., 2018)
miR-133b	SIRT1		(Sun et al., 2018b)
miR-199b			
miR-135a	TRPC1		(He et al., 2014)
miR-184	LPP3		(Zanchi et al., 2017)
miR-370	CNPY1		(Yu et al., 2019)
miR-30c	JAK1; Snail1	Anti-fibrosis	(Zhao et al., 2017; Gao et al., 2020)
miR-98-5p	HMGA2		(Zhu et al., 2019c)
miR-302a-3p	ZEB1		(Tang et al., 2018b)
miR-342	SOX6		(Jiang et al., 2020b)
miR-379-5p	LIN28B		(Li et al., 2019b)
miR-455-3p	ROCK2	Anti-fibrosis	(Wu et al., 2018a; Zhu et al., 2019b)
miR-544	FASN	Anti-inflammation	(Sun et al., 2020)
miR-217	SIRT1/HIF-1α	Pro-fibrosis	(Shao et al., 2016)
		Pro-inflammation	
miR-770-5p	TIMP3	Pro-inflammation	(Zhang et al., 2019c; Wang and Li, 2020)
miR-15b-5p	Sema3A	Anti-inflammation	(Fu et al., 2019)
miR-34b	IL-6R		(Lv et al., 2019)
miR-140-5p	TLR4		(Su et al., 2020)
miR-146a	NOX4		(Wan and Li, 2018)
miR-218	IKK-β		(Li et al., 2020a)
miR-374a	MCP-1		(Yang et al., 2018)
miR-423-5p	NOX4		(Xu et al., 2018c)
miR-451	LMP7		(Sun et al., 2016b)
miR-485	NOX5		(Wu et al., 2020)
miR-874	TLR4		(Yao et al., 2018)

Hyperglycemia triggers the inflammatory response by recruiting immune infiltration and inducing the production of pro-inflammatory cytokines. Of note, podocyte is the barrier to maintain glomerular filtration, and it also functions as the receptor and producer of various cytokines. The dysfunction of podocyte is an essential event in lesion development and glomerulonephritis. This process promotes the progression of DKD (Lal and Patrakka, 2018). Stimulated by HG, miR-770-5p is upregulated and promotes podocyte injury by targeting metalloproteinase 3 (TIMP3), and Tp53 regulated inhibitor of apoptosis 1 (TRIAP1), knocking down of miR-770-5p reverse the apoptosis and inflammation induced by HG in kidney biopsy and mouse podocytes (Zhang et al., 2019c; Wang and Li, 2020). On the other hand, more anti-inflammation related miRNAs have been identified. Overexpression of miR-15b-5p significantly restrained HG-induced apoptosis, oxidative stress and inflammation in podocytes, it also directly targets Sema3A, suggesting that miR-15b-5p could be a therapeutic target for DKD (Fu et al., 2019). miR-34b targets to the interleukin-6 (IL-6) receptor and downstream JAK2/STAT3 signaling, thus reducing the expression of TNF-α, IL-6, interleukin-1β (IL-1β), and caspase-3 in TECs (Lv et al., 2019). The nicotinamide adenine dinucleotide phosphate (NAPDH) oxidase (NOX)-derived reactive oxygen species (ROS) may induce inflammation, implying that NOX enzymes as novel targets for DKD (Lambeth et al., 2008). Of note, miR-146a (Wan and Li, 2018), miR-423-5p (Xu et al., 2018c), and miR-485 (Wu et al., 2020) target NOX4 and NOX5, respectively, to reduce the production of pro-inflammatory cytokines. NF-κB signaling pathway is the classical player in inflammation, which is activated in a wide range of kidney diseases, including DKD. miR-218 targets the IKK-β to regulate NF-κB signaling, as well as reducing the expression of TNF-α, IL-6, IL-1β, and MCP-1 (Li et al., 2020a). miR-451 also targets large multifunctional protease (LMP7) to modulate NF-κB-mediated renal inflammation, which is confirmed by the downregulating level of pro-inflammatory molecules (Sun et al., 2016a). In addition, miR-140-5p and miR-874 also function as anti-inflammatory modulators in suppressing the expression of TNF-α, IL-6, IL-1β in TECs by directly binding to toll like receptor 4 (TLR4), the upstream molecule of NF-κB signaling (Yao et al., 2018; Su et al., 2020). These reports suggest that miRNA-mediated renal fibrosis and inflammation have critical functions in DKD.

### Non-Smad-dependent lncRNAs in DKD

As promising candidates, some miRNA drugs have been approved to proceed toward phase III or IV trials in the coming future. However, the toxicity and off-target effects of miRNA are somehow inevitable (Seok et al., 2018; Hanna et al., 2019). The emerging studies on lncRNAs have shed light on their characteristics of tissue-and-cell-type-specificity and regulation on both transcriptional and translational levels, making lncRNA as the promising therapeutic targets and attractive drugs for DKD treatment **(** Table 2) (Kato, 2018; Guo et al., 2019).

**TABLE 2:** Non-Smad-dependent lncRNAs and their mechanisms in DKD.

lncRNA	Target	Pathological output(s)	References
ZEB1-AS1	miR-216a-5p; MLL1; p53	Anti-fibrosis	(Wang et al., 2018a; Meng et al., 2020)
NR_038323	miR-324-3p; DUSP1		(Ge et al., 2019b)
1700020I14Rik	miR-34a-5p		(Li et al., 2018a)
CYP4B1-PS1-001	Nucleolin		(Wang et al., 2016b; Wang et al., 2018c)
ENSMUST00000147869	*Cyp4a12a*		(Wang et al., 2016c)
XIST	miR-93-5p; CDKN1A	Pro-fibrosis	(Yang et al., 2019a)
PVT1	miR-23b-3p; WT1		(Zhong et al., 2020)
SNHG16	miR-141-3p; CCND1		(Jiang et al., 2020a)
OIP5-AS1	miR-30c-5p		(Fu et al., 2020)
LINC00968	p21/EZH2		(Li et al., 2018b)
ASncmtRNA-2	ROS		(Gao et al., 2017a)
MEG3	miR-181a; Egr-1; TLR4; miR-145	Pro-fibrosis	(Li et al., 2019a; Zha et al., 2019)
BLNC1	NRF2/HO-1; NF-κB		(Feng et al., 2019)
NEAT1	Klotho/ERK1/2; miR-23c; Akt/mTOR; miR-27b-3p/ZEB1		(Ma et al., 2019a; Huang et al., 2019b; Wang et al., 2019b; Li et al., 2020b; Yang et al., 2020)
MALAT1	Wnt/β-catenin; miR-145/ZEB2; SRSF1; IL-6; TNF-α		(Puthanveetil et al., 2015; Hu et al., 2017; Liu et al., 2019a; Zhang et al., 2019a)
Hottip	miR-455-3p; Wnt2B		(Zhu et al., 2019b)
Gm4419	NF-κB/NLRP3; p50		(Yi et al., 2017)
GAS5	MMP9; miR-221; SIRT1	Anti-fibrosis	(Ge et al., 2019a; Zhang et al., 2020)
		Anti-inflammation	
Rpph1	Gal-3/Mek/Erk	Pro-inflammation	(Zhang et al., 2019b)
HOXA-AS2	miR-302b-3p; TIMP3	Anti-inflammation	(Li and Yu, 2020)

LncRNA zinc finger E-box binding homeobox 1 antisense 1 (ZEB1-AS1) plays a protective role in DKD by targeting profibrotic miR-216a-5p to inhibit HK-induced EMT and renal fibrosis. Besides, the anti-fibrotic function of ZEB1-AS1 is also verified that it may bind to H3K4 methyltransferase myeloid and lymphoid or mixed-lineage leukemia 1 (MLL1) and p53 in patients with DKD (Wang et al., 2018a; Meng et al., 2020). lncRNA NR_038323 exerts an anti-fibrotic effect by interacting with miR-324-3p. miR-324-3p is verified to induce dual-specificity protein phosphatase-1 (DUSP1) and the activation of p38/MAPK and ERK1/2 signaling (Ge et al., 2019b). Moreover, the expression of lncRNA 1700020I14Rik is decreased in db/db mice. Bioinformatic method and RNA binding protein immunoprecipitation assay have confirmed the interaction of 1700020I14Rik and miR-34a-5p, which may then modulate the SIRT1/HIF-1α signaling to prohibit renal fibrosis (Li et al., 2018a). Nucleolin is a nuclear protein that expresses on the surface of endothelial cells. CYP4B1-PS1-001 is the lncRNA that upregulated in early DKD. By direct interaction with Nucleolin, CYP4B1-PS1-001 inhibits fibrosis in MCs (Wang et al., 2016b; Wang et al., 2018c). Nevertheless, some lncRNAs interact with miRNAs to trigger and promote the fibrotic process. (Yang et al., 2019a; Jiang et al., 2020a; Fu et al., 2020; Zhong et al., 2020). Interestingly, LINC00968 inhibits p21 by recruiting EZH2 to enhance proliferation and fibrosis of MCs (Li et al., 2018b). ASncmtRNA-2 is upregulated by ROS, and it promotes the expression of TGF-β1 and other fibrotic factors (Gao et al., 2017b).

As shown in Table 2, by direct interaction with miRNAs or inflammatory molecules, lncRNAs play as sponges, inhibitors, or activators to influence either fibrogenesis or inflammatory response. All these findings have demonstrated a critical role of lncRNAs therapeutic targets in the pathogenesis of DKD.

## TGF-β/Smad-dependent Non-coding RNAs in DKD

TGF-β signaling is highly activated under diabetic conditions and has been shown to be a major pathway leading to DKD. It has been well established that DKD-associated fibrosis and inflammation are mediated by TGF-β via Smad-dependent or -independent signaling pathways (Chung and Lan, 2015; Tang et al., 2018a). Active TGF-β1 binds and activates TGF-β receptor II (TβRII) and receptor I (TβRI) which induces phosphorylation of Smad2/3 to form a complex with Smad4 that translocate into the nucleus to regulate transcription of target genes. In general, Smad3 is pathogenic, while Smad2 and Smad7 are protective. Smad4 plays diverse roles in renal fibrosis and inflammation, suggesting Smad4 may not serve as the ideal therapeutic target for DKD (Chung et al., 2013; Li et al., 2014). Many ncRNAs are induced by TGF-β to regulate renal fibrosis and inflammation via Smad-dependent mechanisms in DKD as highlighted in Table 3.

**TABLE 3:** TGF-β/Smad3-dependent miRNAs and lncRNAs in renal fibrotic and inflammatory response of DKD.

miRNA	Mechanism/target	Pathological output(s)	References
miR-192	p53; Zeb1/2; E-cadherin; Egr1	Anti/pro-fibrosis	(Kato et al., 2007; Chung et al., 2010; Krupa et al., 2010; Kato et al., 2011b; Putta et al., 2012; Deshpande et al., 2013; Ma et al., 2016; Liu et al., 2018)
miR-200	TGF-β1/2		(Kato et al., 2011a; Wang et al., 2011)
miR-29c	Spry1; TPM1		(Long et al., 2011; Shao et al., 2019; Huang et al., 2020)
miR-21	Smad7; Spry; PPARα; PTEN; CDC25a; CDK6; MMP9; TIMP1; TIMP3	Pro-fibrosis	(Zhong et al., 2011; Wang et al., 2013; Zhong et al., 2013; Wang et al., 2014; Lai et al., 2015; Mcclelland et al., 2015; Kölling et al., 2017; Chen et al., 2018)
		Pro-inflammation	
miR-27a	SFRP1; PRKAA2; PPARγ	Pro-fibrosis	(Hou et al., 2016; Wu et al., 2018b; Shi et al., 2020)
miR-130b	TGF-β1; Smad2/3; Smad4		(Castro et al., 2014; Lv et al., 2015; Liu et al., 2019b; Ma et al., 2019b)
miR-215	CTNNBIP1		(Mu et al., 2013a)
miR-216a	Ybx1; FoxO1		(Huang et al., 2019a; Meng et al., 2020)
miR-382	HSPD1; FoxO1		(Fang et al., 2017; Wang et al., 2018d)
miR-488	TGF-β1		(Sun et al., 2019)
miR-26a	CTGF; Smad4	Anti-fibrosis	(Koga et al., 2015; Cai et al., 2018; Dong, 2019; Gao et al., 2019)
miR-29a,b	TGF-β1/2; Spry; Col; MMP; Fos; Adams; HDAC4		(Qin et al., 2011; Winbanks et al., 2011; Lan, 2012b; Wang et al., 2012; Chen et al., 2014; Srivastava et al., 2019; Tung et al., 2019)
miR-93	Orai1		(Ma et al., 2018; Yang et al., 2019a; Yang et al., 2019b)
miR-136	SYK; TGF-β/Smad3		(Liu et al., 2020)
miR-let-7	TβR1		(Srivastava et al., 2020)
lncRNA			
Erbb4-IR	miR-29b; Smad7	Pro-fibrosis	(Sun et al., 2018a; Feng et al., 2018; Xu et al., 2020)
NR_033515	miR-743b-5p		(Gao et al., 2018)
Arid2-IR	Egr1; Smad3	Pro-fibrosis	(Zhou et al., 2015; Yang et al., 2019c)
		Pro-inflammation	
NONHSAG053901	Egr-1	Pro-inflammation	(Peng et al., 2019)
LRNA9884	MCP-1		(Zhang et al., 2019d)
TUG1	TGF-β1; PI3K/AKT; miR-21; miR-377; PGC-1α; TRAF5;	Anti-fibrosis	(Li and Susztak, 2016; Long et al., 2016; Duan et al., 2017; Lei et al., 2018; Wang et al., 2019a; Shen et al., 2019; Zang et al., 2019)
PRINS	Smad7	Anti-fibrosis	(Jiao et al., 2019)
		Anti-inflammation	

### TGF-β/Smad-dependent miRNAs in Renal Fibrosis and Inflammation in DKD

miR-192 is the first landmark found in DKD (Kato et al., 2007). TGF-β upregulated miR-192 in MCs and glomeruli from db/db mice, STZ-induced mice model as well as in DKD patients (Kato et al., 2007; Krupa et al., 2010; Putta et al., 2012; Ma et al., 2016; Liu et al., 2018). Indeed, these studies have shown the high correlation between miR-192 and diabetic kidneys. Mechanistically, miR-192 may promote the expression of collagens by targeting the E-box repressor Smad-1 interacting protein (SIP1 or Zeb2) (Kato et al., 2007; Putta et al., 2012). Also, activation of Akt may lead to MCs proliferation and hypertrophy in DKD. miR-192 upregulates miR-216a and miR-217, inhibiting PTEN to induce Akt activation under diabetic conditions. Nevertheless, miR-192 also plays a complex and diverse role in DKD depending on different models or time points. One study has observed a correlation between miR-192 level, tubulointerstitial fibrosis, and eGFR. TGF-β treatment decreases the expression of miR-192 in TECs, resulting in the promotion of fibrosis and the decline of eGFR (Krupa et al., 2010). Similarly, by targeting Egr1, miR-192 decreases the expression of TGF-β1 and fibronectin in glucose-treated TECs and Otsuka-Long-Evans-Tokushima-Fatty rats, a diabetic murine model (Liu et al., 2018). These studies have reported the complexity of miRNA in mediating the fibrotic process in DKD.

miR-200 family (miR-200a, miR-200b, miR-200c) is well-studied miRNA clusters that maintain the epithelial differentiation in cells. Induced by TGF-β or hyperglycemia, the expression of miR-200a are downregulated in TECs. miR-200a functions as a suppressor to EMT, thus protecting kidney from diabetic insults by inhibiting the TGF-β-mediated fibrotic process. Mechanistic study has further revealed that miR-200a downregulates TGF-β2 expression by directly targeting the 3’UTR of TGF-β2 (Wang et al., 2011). However, the expression of miR-200b/c are elevated in glomeruli from type 1 diabetes (T1DM) and type 2 diabetes (T2DM) mice model and in MCs treated with TGF-β1 (Kato et al., 2011b), implying that difference on the miR-200 expression may due to cell type specificity and individual variability. miR-200 family may serve as the therapeutic targets specific to certain cell types response to DKD process.

miR-21 is another well-studied miRNA in renal disease. Although the expression of miR-21 is downregulated in early DKD (Zhang et al., 2009b), it is upregulated in TECs and MCs stimulated by TGF-β1 or HG and in the renal biopsies of DKD patients (Zhong et al., 2011; Wang et al., 2013; Zhong et al., 2013; Wang et al., 2014; Lai et al., 2015; Mcclelland et al., 2015; Kölling et al., 2017; Chen et al., 2018). The mechanism of miR-21 participates in DKD may be related to its activation on both canonical and noncanonical TGF-β signaling. miR-21 not only suppresses the inhibitory Smad7 of TGF-β signaling to promote fibrosis (Zhong et al., 2013; Wang et al., 2014) but also targeting the Sprouty (SPRY) to activate the Ras/MEK/ERK signaling to activate fibrogenesis of TGF-β signaling (Xu et al., 2014). In addition, miR-21 also exerts profibrotic and pro-inflammatory effects by targeting PTEN, tissue inhibitor of matrix metalloproteinases (TIMPs), and other molecules, as shown in Table 3.

miR-29 family is demonstrated to be protective miRNAs that are highly expressed in kidneys but significantly reduced under diabetic conditions. The expression of miR-29 family in various renal cells is decreased when they are stimulated with TGF-β1 or treated with HG (Qin et al., 2011; Chen et al., 2014). The protective role of miR-29 family has been supported by the evidence that overexpression of miR-29 may inhibit the transcription of collagen genes while suppression of miR-29 promotes ECM accumulation. Many studies have identified fibrosis-related targets of miR-29 under hyperglycemic conditions, demonstrating the anti-fibrotic role of miR-29 in DKD. Insterestingly, studies also revealed that miR-29c, serves as a signature miRNA that promotes the progression of DN and fibrosis (Long et al., 2011; Shao et al., 2019; Huang et al., 2020). More and more studies are revealing the functions and mechanisms of miRNAs in fibrosis and inflammation during diabetic conditions, these miRNAs may play as potential therapeutic targets to combat DKD.

### TGF-β/Smad-Dependent lncRNAs in Renal Fibrosis and Inflammation in DKD

Under hyperglycemic condition, the expression of profibrotic and pro-inflammatory lncRNAs are usually upregulated, suggesting their regulatory role in DKD. TGF-β-mediated lncRNA Erbb4-IR is highly expressed in diabetic db/db mice and AGEs-treated MCs. It is regulated by Smad3 as Smad3 deficiency inhibits the transcription of Erbb4 (Feng et al., 2018; Xu et al., 2020). The upregulation of Erbb4-IR is consistent with the elevation of albuminuria, serum creatinine, and fibrotic biomarkers. The mechanistic role of Erbb4-IR may be the binding of Erbb4-IR with the 3’UTR of miR-29b, therefore suppressing anti-fibrotic miR-29b expression. Moreover, Erbb4-IR may also bind with Smad7 to promote renal fibrosis (Sun et al., 2018a; Feng et al., 2018).

lncRNA NR_033515 is found to be significantly increased in the serum of DKD patients, which has shown a positive correlation with KIM-1 and NGAL, diagnostic markers of DKD. The mechanistic study has further confirmed the fibrotic role of NR_033515 by revealing the binding of NR_033515 and miR-743b-5p, resulting in the proliferation, EMT, and fibrosis increasing level of proliferation-related proliferating cell nuclear antigen (PCNA), Cyclin D1, and the fibrotic proteins during DKD (Gao et al., 2018).

Arid2-IR is regulated by Smad3, knockdown of Arid2-IR in TECs has no effect on TGF-β/Smad-mediated fibrosis but promotes IL-1β-induced NF-κB-driven renal inflammation in obstructive kidney disease (Zhou et al., 2015). However, a recent study has reported the profibrotic effect of Arid2-IR by interacting with early growth response protein-1 (Egr1) in high-fat-diet and STZ-induced mice. Arid2-IR induces the expression of collagens and α-SMA in mouse MCs, contributing to the ECM accumulation in DKD (Yang et al., 2019c).

Interestingly, lncRNA NONHSAG053901 also targets Egr1 in mouse MCs, but their interaction has promoted inflammation by upregulating pro-inflammatory cytokines (Peng et al., 2019). The pathogenic role of Smad3-regulated LRNA9884 is observed in db/db mice with more severe albuminuria, histological injuries, and a decline of eGFR. LRNA9884 is induced by AGEs, and it targets MCP-1 to promote MCP-1-driven renal inflammation (Zhang et al., 2019d).

lncRNAs taurine upregulated gene 1 (TUG1) is an anti-fibrotic lncRNA mediated by TGF-β with multiple functions in DKD. In response to metabolic alterations of DKD, the expression of TUG1 is downregulated in podocytes. Overexpression of TUG1 can reverse the mitochondrial dysfunction in podocytes by targeting the transcription factor peroxisome proliferator-activated receptor γ (PPARγ) coactivator 1α (PGC-1α) (Li and Susztak, 2016; Shen et al., 2019). In consistence with previous results, TUG1 can also modulate mitochondrial bioenergetics in podocytes by binding with PGC-1α (Long et al., 2016). These findings have highlighted the connection between lncRNAs and DKD. By interacting with TNF receptor-associated factor 5 (TRAF5), TUG1 can suppress TRAF5-mediated podocyte apoptosis (Lei et al., 2018) and negatively downregulate the PI3K/Akt signaling to inhibit proliferation and ECM deposit in MCs (Zang et al., 2019). TUG1 is also able to interact with miR-21, thus promoting the expression of TIMP3 to alleviate renal fibrosis in HG-stimulated TECs and in db/db mice (Wang et al., 2019a). Furthermore, TUG1 sponges for miR-377 to regulate PPARγ and ECM in MCs (Duan et al., 2017). All these protective effects of lncRNA TUG1 in various cell types has supported its therapeutic potential in treating DKD. Besides, some lncRNAs may play diverse roles in the pathogenesis of DKD. lncRNA psoriasis-susceptibility related RNA gene induced by stress (PRINS) may exert both anti-fibrotic, anti-inflammatory but pro-apoptotic effects by regulating Smad7 in DKD. It has been demonstrated that there is a positive correlation between PRINS and Smad7 in DKD patients. As overexpression of Smad7 inhibits renal fibrosis and inflammation but also induces apoptosis in podocytes (Schiffer et al., 2001; Ka et al., 2012), thus, overexpression of PRINS upregulates Smad7 expression and promotes apoptosis in mouse podocytes (Jiao et al., 2019). lncRNA PRINS may be a therapeutic target of DKD-induced renal fibrosis and inflammation. But the underlying mechanisms of interaction between PRINS and Smad7 remain unexplored. In conclusion, the connection of TGF-β-mediated lncRNA and DKD is well-defined. Further studies on revealing the therapeutic targets and underlying mechanisms of these lncRNAs remain to be further explored.

## Non-coding RNAs as Novel Biomarkers for DKD

The diagnosis and monitoring of renal injuries in DKD are now dependent on the detection of urinary albumin or serum creatinine. However, some patients may not present microalbuminuria or creatinine alterations during the progression of DKD, suggesting that none of these measures can accurately indicate the severity and type of injury induced by hyperglycemia (Magee et al., 2017; Lin et al., 2018). In addition, urinary albumin is not specific to DKD, which may also occur in other diseases. Besides, the diagnostic and prognostic test of renal biopsy is invasive and may not be a reliable way to establish the full patterns of DKD. Thus, the availability of sensitive and specific biomarkers will provide therapeutic benefits in the control of DKD.

Non-coding RNAs in body fluids could facilitate communication between cells. Non-coding RNAs may exist in a stable form in serum and urine. As biomarkers, they may form a complex with proteins or be stored in transporters, including exosomes, microparticles, and apoptotic bodies. Based on the tissue- and cell type-specific characteristics of lncRNAs, significant differences in expression of novel lncRNAs in DKD (as shown in Tables 2 and 3) have mapped the signaling pathways in the pathogenesis of diabetic nephropathy (Guo et al., 2019). Indeed, a recent study has reported a novel lncRNA, PANDAR, related to T2DM DKD patients. The expression of PANDAR is upregulated in diabetic patients and higher in DKD patients with massive proteinuria, demonstrating its potential as biomarker and predictor for prognosis and progression of DKD (Zhao et al., 2020). The expression of lncRNA CASC2 is downregulated in T2DM patients with chronic renal failure but not T2DM patients with other complications, suggesting that lncRNA cancer susceptibility candidate 2 (CASC2) could also serve as a renal specific biomarker for DKD. Moreover, the study has further followed up for 5 years and found out that serum level of lncRNA CASC2 is negatively correlated with the incidence of chronic renal failure, supporting that serum level of lncRNA CASC2 may be a specific and reliable biomarker for diagnosis in DKD progression (Wang et al., 2018b). These studies have shown that lncRNAs are of high relevance in the development and progression of DKD, however, further mechanistic investigations on lncRNAs as therapeutic targets are warranted.

Some circulating miRNAs may also serve as sensitive and useful biomarkers for early detection and diagnosis for DKD (Zhang et al., 2016; Nascimento and Domingueti, 2019). For instance, in the early stage of T2DM DKD, the expression of miR-377 is positive, while miR-192 is negatively correlated with renal function (Tayel et al., 2020). In addition, circulating miRNA of miR-1246, miR-642a-3p, let-7c-5p, miR-1255b-5p, let-7i-3p, miR-5010-5p, and miR-150-3p are significantly upregulated in DKD patients compared with healthy volunteers (Kim et al., 2019). Moreover, the expression of miR-126 is decreased in DKD patients, which is negatively associated with albuminuria, level of fasting glucose, and glycated hemoglobin but positively correlated with eGFR (Al-Kafaji et al., 2016). The level of serum miR-21 is also consistent with tissue miR-21 that closely reflects renal function in DKD (Wang et al., 2016a). Up to date, many studies have reported the expression profiles of circulating miRNAs in diabetic nephropathy, making miRNAs as one of the promising candidates for DKD diagnosis and therapeutic targets.

The urinary exosomal miRNAs are called as “liquid biopsy” (La Marca and Fierabracci, 2017), which are typically secreted by cells from renal segments. They carry proteins, RNAs, and biomarkers that may reflect renal injury and dysfunction (Xu et al., 2018b). For example, miR-200b is a novel urinary biomarker that negatively correlates with the degree of renal fibrosis in CKD and DKD (Yu et al., 2018). One study has suggested that the expression of miR-27b-3p and miR-1228-3p in urine may be useful indicators for the degrees of renal fibrosis of DKD patients (Conserva et al., 2019). Notably, the expression of miR-126 in urine is increased in DKD patients compared to diabetic patients without renal disease. Interestingly, the urinary level of miR-126 is significantly decreased in DKD patients with a better diabetic control, implying that miR-126 may be a biomarker in DKD and monitor for DKD treatment response (Liu et al., 2014).

Although the clinical relevance in urinary miRNAs have been well studied (Lv et al., 2013; Cheng et al., 2014), there is still no consensus on the normalization of miRNAs isolated from urine, as the levels of urinary miRNAs may be high veriable and affected by urinary contents and concentrations. Neverthless, the better normalizer strategies should be encouraged (Blondal et al., 2013; Lekchnov et al., 2016; Corral-Vazquez et al., 2017), as the normalization of the validated data may help to provide statistically significant results without causing unwanted bias.

## Non-coding RNAs as Promising Therapeutic Targets for DKD

**FIGURE 3:**
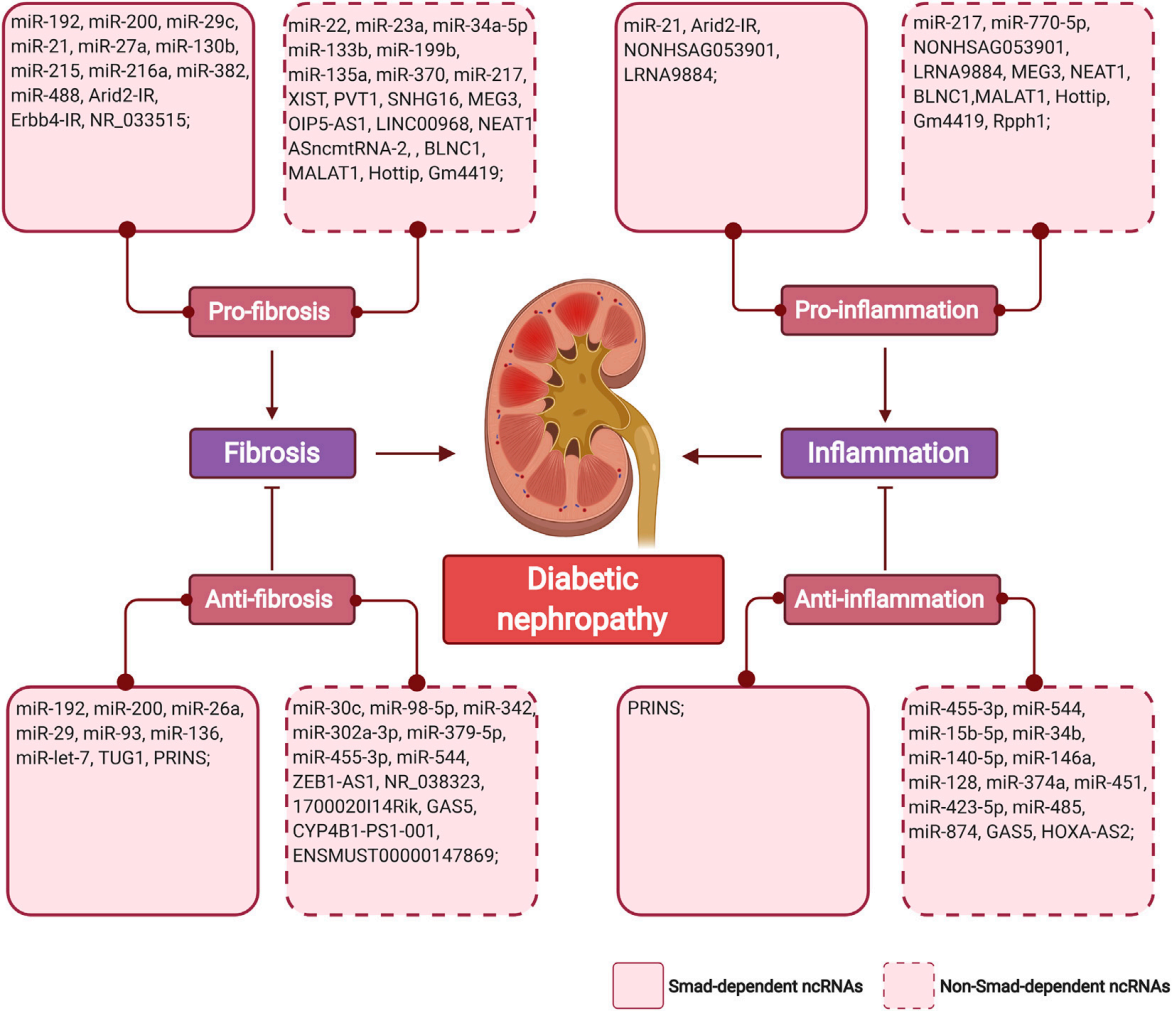
The summary of TGF-β/Smad-dependent and non-TGF-β/Smad-dependent miRNAs and lncRNAs in diabetic renal fibrosis and inflammation. Non-coding RNAs are classified as pro/anti-fibrosis, pro/anti-inflammation in regard with their mechanistic funcions in diabetic nephropathy. (Figure created with BioRender.com).

The regulatory role of non-coding RNAs in the pathogenesis of DKD has highlighted their potential as therapeutic targets for DKD. Restoring expression or inhibition of non-coding RNAs in renal or inflammatory cells under diabetic conditions may halt renal fibrosis and inflammation (Figure 3). Besides, rebalancing the overactivated TGF-β signaling induced by hyperglycemia could be another strategy that controls renal complication.

The delivery of synthetic non-coding RNA oligonucleotides, plasmids, or inhibitors may alter pathogenic signaling pathways related to DKD. Antagonism of miR-21 not only reduces the loss of podocytes and albuminuria but also inhibits renal fibrotic response by inhibition of collagen and fibronectin *in vivo* and *in vitro* (Wang et al., 2013; Kölling et al., 2017; Roy et al., 2020). Silencing miR-215 with specific antagomir increases the expression of CTNNBIP1, reduces of β-catenin activity, and accumulation of fibrotic proteins in db/db mice (Mu et al., 2013b). We have established the non-invasive ultrasound microbubble-mediated gene transfer to knock down renal expression of miR-21, thus suppressing the activation of the TGF-β and NF-κB signaling pathways by targeting Smad7 in the diabetic mouse model (Zhong et al., 2013). In addition, restoring the expression of miR-29b by delivery of doxycycline-inducible pre-miR-29b into the kidney, could significantly reverse the pathological changes of progressive DKD (Chen et al., 2014). Moreover, kidney-specific silencing of lncRNA Erbb4-IR and LRNA9884 with ultrasound technique can convert plasmids into the damaged kidney to ameliorate injuries, albuminuria, fibrosis, and inflammation (Sun et al., 2018a; Zhang et al., 2019d). Notably, exosomes secreted by cells contain non-coding RNAs that may have a regulatory role in DKD. Injection of exosomes from HG-treated macrophages induces MCs proliferation, fibrotic, and inflammatory factors activation *in vivo* as well as *in vitro*. Intriguingly, exosomes from TGF-β1 knockdown macrophages may reverse pathogenic changes in MCs (Zhu et al., 2019a), underscoring the importance of TGF-β signaling in the pathogenesis of DKD.

The rapid development of the field of non-coding RNAs has helped these RNA-based biopharmaceuticals to enter clinical trials before market approval. However, non-coding RNA treatments remain to be explored. The low expression, low conservation between species, time specificity, toxicity, and off-target effect of non-coding RNA are obstacles waiting to be solved in the development of RNA therapy (Yang et al., 2014; Ard et al., 2017). Up to date, the number of non-coding RNAs related to clinical trials on DKD is limited (Sankrityayan et al., 2019). Nevertheless, some ongoing miRNA-based therapies may be the potential next-generation medicine for DKD (Chakraborty et al., 2017). For example, Remlarsen, a miR-29 mimic that is undergoing in the clinical test (https://clinicaltrials.gov/ct2/show/NCT03601052) and could be the promising drug to combat renal fibrosis in DKD. Hopefully, new technologies such as clustered regularly interspaced short palindromic repeats (CRISPR) and CRISPR-associated (Cas) gene editing may represent novel strategies to modulate the expression and function of non-coding RNAs in DKD (Miano et al., 2019). Further studies are needed to reveal the therapeutic potential of ncRNAs in the clinical treatment of DKD.

## Conclusion and Future Perspectives

Non-coding RNAs have garnered the major attention of researchers in the past few decades. We are now shifting toward their regulatory role and mutual relationship in the pathogenesis of DKD. Reports in this review and available literature have drawn the patterns of ncRNAs profiles in the process of diabetic nephropathy, but further investigation into the crucial mechanisms of ncRNAs in epigenetic regulation is warranted. Moreover, as biomarkers, the expression of renal ncRNAs may reflect the cellular response to hyperglycemic injuries, thus contributing to the early diagnosis and prognosis of DKD. The discovery of miRNAs and lncRNAs also represents a new field of molecular therapy into DKD treatment. Together these findings are expected to yield novel insights into the complex pathogenesis of DKD and could be incorporated in the clinical settings.

## Author Contributions

Y-YG wrote and revised the manuscript. F-HL, X-RH, WM, and LZ revised the manuscript. X-SL, X-QY, and H-YL revised and edited the manuscript. All authors contributed to the manuscript conception development, data collection and analysis, and discussion on the manuscript writing and revising.

## Funding

This work was supported by the Guangdong-Hong Kong-Macao-Joint Labs Program from Guangdong Science and Technology (2019B121205005); the Research Grants Council of Hong Kong (Grants GRF 14163317, 14117418, 14104019, R4012-18, and C7018-16G); Lui Che Woo Institute of Innovative Medicine (CARE); the Health and Medical Research Fund of Hong Kong (Grants HMRF 05161326, 06173986, and 14152321); the National Natural Science Foundation of China (No.81873261 and No. 81903956), the Project of Guangdong Province Administration of Traditional Chinese Medicine (No. 20201133). Project from the State Key Laboratory of Dampness Syndrome of Chinese Medicine (SZ2020ZZ22).

## Conflict of Interest

The authors declare that the research was conducted in the absence of any commercial or financial relationships that could be construed as a potential conflict of interest.
